# Inequalities in access to and outcomes of cardiac surgery in England: retrospective analysis of Hospital Episode Statistics (2010–2019)

**DOI:** 10.1136/heartjnl-2024-324292

**Published:** 2024-09-03

**Authors:** Florence Y Lai, Ben Gibbison, Alicia O’Cathain, Enoch Akowuah, John G Cleland, Gianni D Angelini, Christina King, Gavin J Murphy, Maria Pufulete

**Affiliations:** 1 Department of Cardiovascular Sciences, University of Leicester, Leicester, UK; 2 NIHR Leicester Biomedical Research Unit in Cardiovascular Medicine, University of Leicester, Leicester, UK; 3 Cardiac Anaesthesia and Intensive Care, Bristol Medical School, University of Bristol, Bristol, UK; 4 Sheffield Health Centre for Health and Related Research, The University of Sheffield, Sheffield, UK; 5 Department of Cardiac Surgery, the James Cook University Hospital, South Tees Hospitals NHS Foundation Trust, Middlesbrough, UK; 6 Robertson Centre for Biostatistics and Clinical Trials, Institute of Health & Wellebing, University of Glasgow, Glasgow, UK; 7 Department of Cardiac Surgery, Bristol Heart Institute, University of Bristol, Bristol, UK; 8 Bristol Heart Institute, University of Bristol, Bristol, UK; 9 Bristol Heart Institute, Bristol Medical School, University of Bristol, Bristol, UK

**Keywords:** Cardiac surgery, Cardiac Surgical Procedures, Coronary Artery Bypass, Heart Valve Prosthesis Implantation, Electronic Health Records

## Abstract

**Background:**

We aimed to characterise the variation in access to and outcomes of cardiac surgery for people in England.

**Methods:**

We included people >18 years of age with hospital admission for ischaemic heart disease (IHD) and heart valve disease (HVD) between 2010 and 2019. Within these populations, we identified people who had coronary artery bypass graft (CABG) and/or valve surgery, respectively. We fitted logistic regression models to examine the effects of age, sex, ethnicity and socioeconomic deprivation on having access to surgery and in-hospital mortality, 1-year mortality and hospital readmission.

**Results:**

We included 292 140 people, of whom 28% were women, 11% were from an ethnic minority and 17% were from the most deprived areas. Across all types of surgery, one in five people are readmitted to hospital within 1 year, rising to almost one in four for valve surgery. Women, black people and people living in the most deprived areas were less likely to have access to surgery (CABG: 59%, 32% and 35% less likely; valve: 31%, 33% and 39% less likely, respectively) and more likely to die within 1 year of surgery (CABG: 24%, 85% and 18% more likely, respectively; valve: 19% (women) and 10% (people from most deprived areas) more likely).

**Conclusions:**

Female sex, black ethnicity and economic deprivation are independently associated with limited access to cardiac surgery and higher post-surgery mortality. Actions are required to address these inequalities.

**WHAT IS ALREADY KNOWN ON THIS TOPIC**

- There has been a marked improvement in short-term (in hospital) outcomes of people having cardiac surgery. It is not clear if this translates to improvement in mid-term and long-term outcomes and how these differ by demographic and socioeconomic factors.

**WHAT THIS STUDY ADDS**

- Across all types of cardiac surgery, one in five people are readmitted to the hospital within 1 year of surgery, rising to almost one in four for valve surgery.
- While in-hospital mortality has decreased markedly since 2010 (by 20%), 1-year mortality and hospital readmission remained largely unchanged.
- Women, people of black ethnicity and people from the most socially deprived areas are less likely to be offered cardiac surgery and more likely to die and be readmitted to hospital in the year after surgery.

**HOW THIS STUDY MIGHT AFFECT RESEARCH, PRACTICE OR POLICY**

- Targeted interventions are required across the cardiovascular medicine pathway to improve mid-term and long-term outcomes in people having cardiac surgery, addressing inequalities.

## Introduction

Cardiac surgery is high-volume surgery, with about 28 000 adults a year undergoing a cardiac surgical procedure in the UK.^1^ It is one of the costliest interventions carried out to treat cardiovascular disease by the UK National Health Service (NHS).^2^ While the National Adult Cardiac Surgery Audit (NACSA) has shown a steady improvement in short-term outcomes of people having cardiac surgery, mid-term and long-term outcomes are unknown.

The influence of social determinants of health (eg, gender, ethnicity and deprivation) on mid-term and long-term outcomes of cardiac surgery has not been explored. It is known that women and people from ethnic minorities and of low socioeconomic status have worse short-term outcomes after all types of cardiac surgery (in-hospital),^3^,^7^ but it is unclear whether these characteristics also translate to poorer mid-term (1 year) and long-term outcomes (3 and 5 years, respectively).

Our study, using the Hospital Episode Statistics (HES) and UK Office for National Statistics (ONS) data, has two objectives: to describe mid-term and long-term outcomes after cardiac surgery, previously not described in the UK, and to characterise the variation in utilisation and outcome of cardiac surgery by sex, ethnicity and deprivation, while also examining intersections between these characteristics.

## Methods

### Study design

Retrospective cohort study using HES (England) linked with ONS mortality data.

### Data sources

HES covers all admissions to NHS hospitals or independent providers that are funded by NHS.^8^ Each anonymised record contains demographics (age, sex, ethnicity, area of residence, index of multiple deprivation (IMD)), administrative (admission and discharge dates, admission method, discharge destination, etc) and clinical information (diagnoses and procedures performed). Diagnoses were coded based on the International Classification of Diseases, Tenth Revision (ICD-10), and procedures were coded by the Office of Population Censuses and Surveys Classification of Interventions and Procedures (OPCS-4). Our dataset comprised all hospital admissions for two cardiovascular diseases: ischaemic heart disease (IHD) and heart valve disease (HVD), which require cardiac surgery as treatment in England, between April 2010 and March 2019 (financial years in the NHS in England).

### Study populations

All adults (>18 years) who had at least one hospital admission (not including day cases) with an IHD diagnosis (ICD10 I20–I25) and an HVD diagnosis (ICD10 I01, I05–I08, I33–I39, I511, I512), respectively, in each financial year during 2010 to 2019. Within these populations, we further identified people who had cardiac surgery defined as coronary artery bypass graft (CABG, OPCS-4 K40–K46) and/or valve surgery OPCS-4 K25–K31, K34, K36.1, K36.2). When examining the outcomes of people undergoing cardiac surgery, we included each person’s first episode of cardiac surgery in the study period and excluded those who had undergone cardiac surgery in the previous 2 years. The full list of ICD-10 and OPCS-4 codes used to define the study populations is shown in online supplemental table S1.

Self-reported ethnicity was grouped as white (British, Irish and other white background), black (Caribbean, African and other black background), South Asian (Bangladeshi, Indian, Pakistani) and others (mixed and other ethnic background). Socioeconomic status was defined using area-level IMD 2015^9^ scores. IMD quintiles were created by dividing the deprivation scores of individual areas into fifths ranging from the 20% most deprived areas to the 20% least deprived areas.

In order to adjust for pre-existing poor health, we identified comorbidities (Royal College of Surgeons (RCS) Charlson Score^10^ based on diagnoses recorded within 1 year prior to the index admission) and frailty status^9^ (defined as occurrence of one or more of seven domains associated with frailty using diagnoses recorded in all hospital admissions within 2 years before the index admission) (online supplemental tables S2 and S3).

### Study outcomes

For the IHD and HVD populations, we calculated the utilisation of cardiac surgery, defined as the proportion of people with IHD who underwent CABG surgery and the proportion of people with HVD who underwent heart valve surgery.

For the cardiac surgery population, we calculated mortality (in-hospital and at 1, 3 and 5 years) and hospital readmission for cardiovascular causes, heart failure and stroke/transient ischaemic attack (TIA). Readmission for cardiovascular causes was defined as admission to any NHS hospital post-cardiac surgery with a cardiovascular disease (ICD10 I00–I99) as the primary diagnosis. Readmission for heart failure and stroke/TIA was defined using both primary and secondary diagnoses.

### Missing data

For age and sex, there were <0.1% missing or invalid entries which were excluded from the analysis. For ethnicity and socioeconomic deprivation (IMD), missing data were imputed if people had valid ethnicity entries from other episodes and valid IMD scores from other episodes within a year from their index episode. If people had multiple episodes that could be used for imputation, the one closest to the index episodes based on episode start date was used. After imputation, the missingness of ethnicity decreased from 6.6% to 1.7% for the IHD population and 7% to 1.8% for the HVD population. For the IMD index, missingness decreased from 0.9% to 0.7% for the IHD population and 1.2% to 0.9% for the HVD population. The RCS Charlson index, frailty status and all outcomes were defined based on ICD10 diagnosis codes. People without the relevant diagnosis codes were treated as absence of the disease, and therefore, these variables have no missing data.

### Statistical analysis

The characteristics of the study population were summarised using descriptive statistics (numbers and percentages; mean and SD). To describe access to surgery over time in the IHD and HVD populations, we calculated rates of CABG surgery per 1000 people with IHD and rates of valve surgery per 1000 people with HVD, standardised by age, Charlson index and frailty status using the respective patient populations in 2019 as the standard population. We examined standardised rates separately for the following subgroups: CABG surgery and valve surgery, by sex, ethnic group and IMD quintile. We fitted two logistic regression models to examine the effects of age, sex, ethnicity and IMD (adjusted for covariates including Charlson comorbidities, frailty status, year of surgery) on having access to CABG in the IHD population and having access to valve surgery in the HVD population.

To assess outcomes from surgery, we separated the cohorts into 2010–2014 and 2015–2019. We used the earlier cohort for calculating 1-year, 3-year and 5-year outcomes and the later cohort for calculating 1-year outcomes. In-hospital mortality and 1-year outcomes were analysed as binary (binomial distribution). We fitted logistic regression models with logit link function for in-hospital mortality and 1-year outcomes only to examine the effects of sex, ethnicity and IMD adjusted for age, Charlson index, frailty status, year of surgery and operative characteristics (scheduled/emergency surgery and whether the surgery involved cardiopulmonary bypass). All variables were entered into the logistic models with no variable selection performed. Models were fitted separately for people undergoing CABG alone, valve surgery alone and combined CABG and valve surgery.

For all the models, we checked for collinearity by examining parameter estimates and their SEs and the correlations among the parameter estimates of the fitted model. We did not find large SEs or high correlations between variables in any of the fitted models.

## Results

The characteristics of the 2010–2014 and 2015–2019 populations are shown in table 1. In 2010–2014, there were 143 104 individuals (54% isolated CABG, 32% isolated valve surgery and 14% combined CABG and valve). In 2015–2019, there were 149 036 individuals (45% isolated CABG, 43% isolated valve surgery and 12% combined CABG and valve). Standardised rates of both CABG and valve surgery decreased between 2010–2019 (online supplemental figure S1), from 30.1 to 24.8 per 1000 people with IHD and from 87.9 to 69.9 per 1000 people with HVD, respectively. The demographics of the two populations (age, sex, ethnicity, socioeconomic quintile) were similar, although the proportions of people with multi-morbidity and frailty and emergency admissions were higher in 2015–2019, across all types of surgery reflecting the increasing comorbidity and frailty over time in the admitted IHD and HVD populations (online supplemental tables S4 and S5).

**Table 1:** Characteristics of people who underwent cardiac surgery across two time periods (2010–2014 and 2015–2019)

	2010–2014					2015–2019				
	CABG alone	Valve surgery alone	Aortic valve	Mitral valve	Concomitant CABG and valve surgery	CABG alone	Valve surgery alone	Aortic valve	Mitral valve	Concomitant CABG and valve surgery
Number of people	76 604	46 308	30 397	9331	20 192	67 416	63 491	45 094	10 785	18 129
Age 18–44	1553 (2%)	3490 (8%)	1397 (5%)	994 (11%)	116 (1%)	1227 (2%)	3738 (6%)	1549 (3%)	1008 (9%)	107 (1%)
45–54	8773 (11%)	3873 (8%)	1941 (6%)	1257 (13%)	641 (3%)	7355 (11%)	4714 (7%)	2465 (5%)	1387 (13%)	627 (3%)
55–64	20 822 (27%)	7799 (17%)	4595 (15%)	2151 (23%)	2609 (13%)	18 891 (28%)	9045 (14%)	5479 (12%)	2351 (22%)	2412 (13%)
65–74	28 038 (37%)	13 698 (30%)	8925 (29%)	2888 (31%)	7233 (36%)	25 622 (38%)	17 570 (28%)	11 877 (26%)	3457 (32%)	6880 (38%)
75+	17 418 (23%)	17 448 (38%)	13 539 (45%)	2041 (22%)	9593 (48%)	14 321 (21%)	28 424 (45%)	23 724 (53%)	2582 (24%)	8103 (45%)
Sex—male	62 457 (82%)	26 245 (57%)	17 571 (58%)	5332 (57%)	14 635 (72%)	55 666 (83%)	37 011 (58%)	26 613 (59%)	6395 (59%)	13 779 (76%)
Ethnicity										
White	66 759 (88%)	43 070 (94%)	28 816 (95%)	8409 (91%)	18 965 (94%)	56 290 (86%)	57 806 (93%)	41 851 (95%)	9382 (89%)	16 779 (94%)
Black	662 (1%)	619 (1%)	278 (1%)	164 (2%)	108 (1%)	655 (1%)	948 (2%)	501 (1%)	221 (2%)	128 (1%)
South Asian	5302 (7%)	1028 (2%)	498 (2%)	301 (3%)	546 (3%)	5291 (8%)	1483 (2%)	852 (2%)	374 (4%)	482 (3%)
Mixed/others	3242 (4%)	1288 (3%)	621 (2%)	392 (4%)	457 (2%)	3408 (5%)	1968 (3%)	1074 (2%)	519 (5%)	445 (2%)
Socioeconomic quintile										
Q1 most deprived	14 113 (19%)	6992 (15%)	4396 (15%)	1422 (16%)	2985 (15%)	12 617 (19%)	9185 (15%)	6362 (14%)	1481 (14%)	2572 (14%)
Q2	14 996 (20%)	8143 (18%)	5365 (18%)	1587 (17%)	3495 (18%)	12 792 (19%)	10 947 (18%)	7834 (18%)	1768 (17%)	3164 (18%)
Q3	15 786 (21%)	9399 (21%)	6359 (21%)	1809 (20%)	4335 (22%)	13 848 (21%)	13 141 (21%)	9422 (21%)	2234 (21%)	3815 (21%)
Q4	15 657 (21%)	10 312 (23%)	6856 (23%)	2093 (23%)	4401 (22%)	14 026 (21%)	14 303 (23%)	10 266 (23%)	2491 (24%)	4146 (23%)
Q5 least deprived	14 680 (20%)	10 367 (23%)	6702 (23%)	2229 (24%)	4505 (23%)	12 998 (20%)	14 619 (24%)	10 350 (23%)	2596 (25%)	4073 (23%)
Emergency admission	12 761 (17%)	4334 (9%)	2836 (9%)	709 (8%)	2225 (11%)	16 316 (24%)	8067 (13%)	5874 (13%)	1057 (10%)	2712 (15%)
Cardiopulmonary bypass	61 350 (80%)	38 204 (82%)	23 612 (78%)	8511 (91%)	19 344 (96%)	56 661 (84%)	43 756 (69%)	26 693 (59%)	9895 (92%)	17 672 (97%)
Charlson index (RCS)										
0	20 213 (26%)	10 931 (24%)	6931 (23%)	2739 (29%)	3851 (19%)	12 619 (19%)	10 237 (16%)	6702 (15%)	2382 (22%)	2480 (14%)
1	25 996 (34%)	15 726 (34%)	10 167 (33%)	3414 (37%)	6163 (31%)	20 658 (31%)	18 794 (30%)	12 867 (29%)	3710 (34%)	4827 (27%)
2	17 229 (22%)	11 250 (24%)	7397 (24%)	2033 (22%)	5263 (26%)	17 191 (25%)	16 980 (27%)	11 995 (27%)	2791 (26%)	4947 (27%)
3+	13 166 (17%)	8401 (18%)	5902 (19%)	1145 (12%)	4915 (24%)	16 948 (25%)	17 480 (28%)	13 530 (30%)	1902 (18%)	5875 (32%)
Frailty syndrome	13 573 (18%)	11 322 (24%)	7701 (25%)	1975 (21%)	4914 (24%)	16 363 (24%)	21 463 (34%)	15 793 (35%)	3140 (29%)	5700 (31%)

CABG, coronary artery bypass graftingQquintileRCSRoyal College of Surgeons

### Access to cardiac surgery

**Figure 1:**
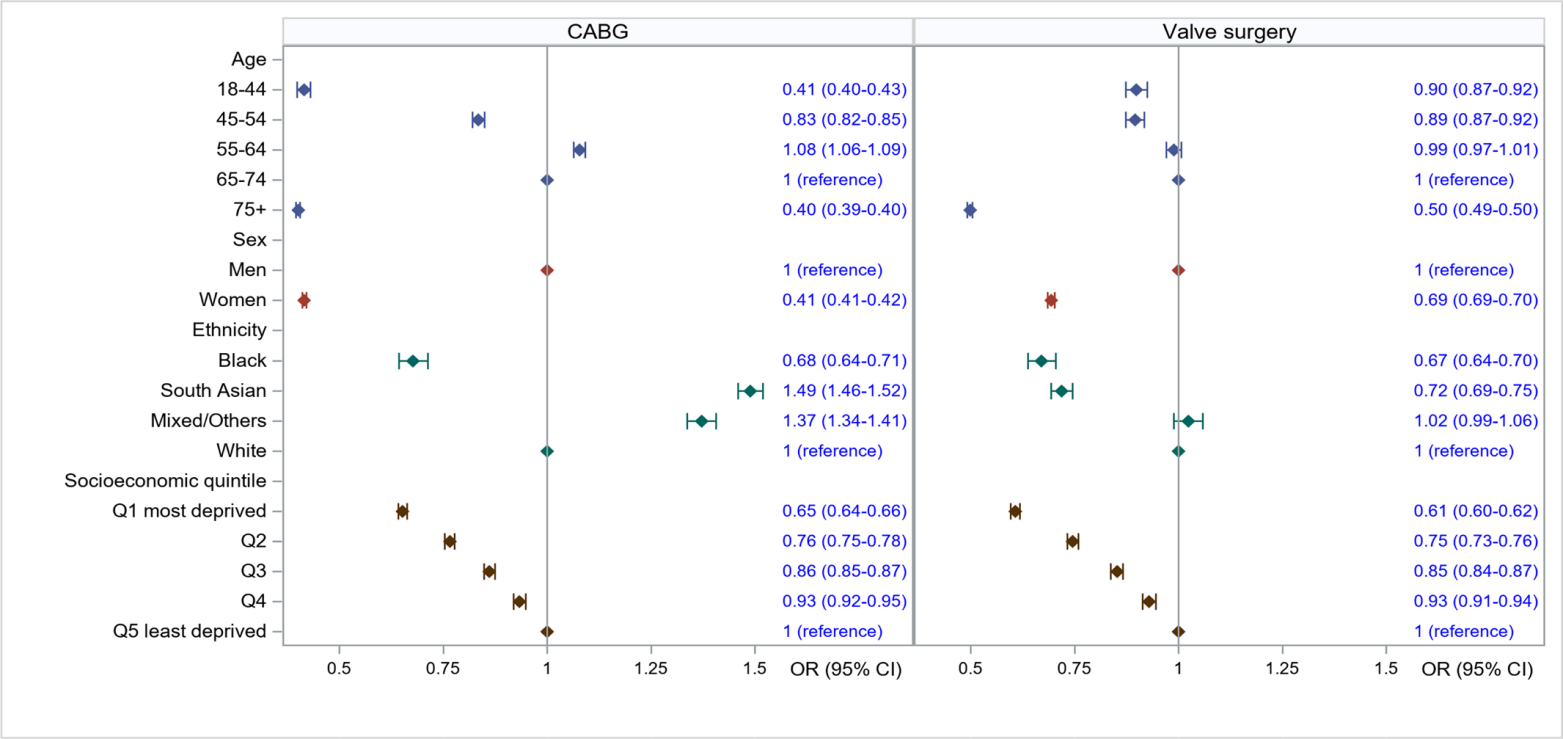
Association of age, sex, ethnicity and socioeconomic quintile on having access to (left) CABG in people with IHD and (right) valve surgery in people with HVD. All models are adjusted for age, Charlson index, frailty status, year of surgery, and operative characteristics (scheduled/emergency surgery, and whether the surgery involved cardiopulmonary bypass). CABG, coronary artery bypass grafting; IHD, ischaemic heart disease; HVD, heart valve disease.

The adjusted associations between age, sex, ethnicity and socioeconomic quintile on access to CABG and valve surgery are shown in figure 1. Women were less likely to have CABG and valve surgery than men (OR 0.41, 95% CI 0.41 to 0.42 and OR 0.69, 95% CI 0.69 to 0.70). Compared with white people, black people were less likely to have surgery (CABG: OR 0.68, 95% CI 0.64 to 0.71; valve: OR 0.67, 95% CI 0.64 to 0.70), while South Asian people were more likely to have CABG (OR 1.49, 95% CI 1.46 to 1.52) but not valve surgery (OR 0.72, 95% CI 0.69 to 0.75). For both CABG and valve surgery, there was almost a linear association between increasing levels of deprivation and decreasing odds of getting surgery, with people in the most deprived group being 35% and 39% less likely to have CABG and valve surgery, respectively, than people in the least deprived group (OR 0.65, 95% CI 0.64 to 0.68 and OR 0.61, 95% CI 0.60 to 0.62). For intersectionality (investigating the combined effects of different inequality factors), standardised rates of both CABG and valve surgery were lowest in black women and women from the lowest socioeconomic quintile, with little change to this pattern between 2010 and 2019 (online supplemental figure S2).

### Outcomes from cardiac surgery

#### In-hospital mortality (short-term)

In-hospital mortality decreased across all types of cardiac surgery between 2010–2014 and 2015–2019 and was lowest in CABG (1.7% and 1.4%, respectively) and highest in combined CABG and valve surgery (5.4% and 4.5%, respectively, table 2).

**Table 2:** Outcomes of people undergoing CABG and valve surgery in 2010–2019

	2010–2014					2015–2019				
	CABG alone	Valve surgery alone	Aortic valve	Mitral valve	Concomitant CABG and valve surgery	CABG alone	Valve surgery alone	Aortic valve	Mitral valve	Concomitant CABG and valve surgery
No of people	76 604	46 308	30 397	9331	20 192	67 416	63 491	45 094	10 785	18 129
In-hospital mortality	1.7%	3.0%	2.5%	2.6%	5.4%	1.4%	2.3%	1.8%	2.0%	4.5%
Mortality (all-cause)										
1 year	3.8%	7.6%	7.5%	5.4%	10.5%	3.4%	7.5%	7.8%	4.9%	9.1%
3 years	7.6%	14.7%	15.8%	9.4%	17.8%					
5 years	12.6%	22.7%	25.2%	13.9%	26.8%					
Cardiovascular-cause hospital readmission										
1 year	17.3%	25.4%	23.6%	27.0%	26.6%	15.5%	23.4%	22.1%	25.1%	24.0%
3 years	27.2%	34.5%	32.8%	35.6%	36.3%					
5 years	34.7%	41.3%	39.9%	41.6%	43.7%					
Hospital admission for heart failure										
1 year	6.4%	11.7%	11.2%	10.4%	13.0%	8.0%	14.9%	14.7%	13.5%	14.8%
3 years	10.5%	18.4%	18.1%	16.2%	19.9%					
5 years	15.0%	24.3%	24.2%	21.0%	26.5%					
Hospital admission for stroke/TIA										
1 year	2.1%	3.4%	3.5%	3.0%	4.0%	2.0%	3.8%	4.1%	2.9%	4.2%
3 years	4.0%	5.9%	6.2%	5.3%	6.8%					
5 years	5.9%	8.1%	8.5%	7.0%	9.3%					

CABG, coronary artery bypass graftingTIAtransient ischaemic attack

**Figure 2:**
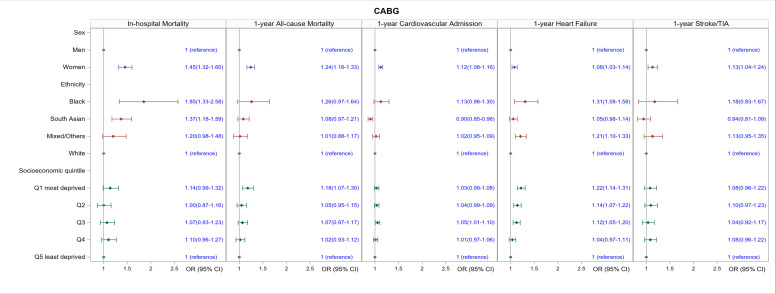
Association of sex, ethnicity and socioeconomic quintile with in-hospital mortality and 1-year outcomes for people undergoing coronary artery bypass grafting (CABG). All models are adjusted for age, Charlson index, frailty status, year of surgery and operative characteristics (scheduled/emergency surgery and whether the surgery involved cardiopulmonary bypass).

Across all types of surgery, women had a higher in-hospital mortality rate than men (CABG: OR 1.45, 95% CI 1.32 to 1.60; valve surgery: OR 1.31, 95% CI 1.21 to 1.4; CABG and valve surgery: OR 1.50, 95% CI 1.36 to 1.66, table 2 and figures2^4^). In terms of ethnicity, South Asian people had a higher in-hospital mortality compared with white people (CABG: OR 1.37, 95% CI 1.18 to 1.59, valve surgery: OR 1.53, 95% CI 1.23 to 1.89; CABG and valve surgery: OR 1.67, 95% CI 1.33 to 2.10). Black people had higher in-hospital mortality than white people after CABG (OR 1.85, 95% CI 1.33 to 2.58), but not valve or CABG and valve surgery. Although crude in-hospital mortality was higher in people with the highest socioeconomic deprivation versus those with the lowest socioeconomic deprivation across all types of surgery (table 2), these differences were not significant after adjustment (figures2^4^).

**Figure 3:**
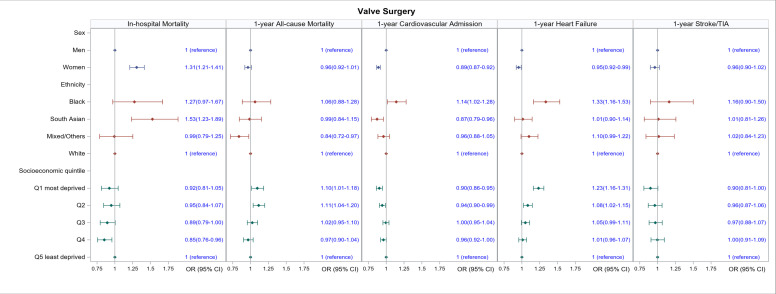
Association of sex, ethnicity and socioeconomic quintile with in-hospital mortality and 1-year outcomes for people undergoing alone valve surgery. All models are adjusted for age, Charlson index, frailty status, year of surgery and operative characteristics (scheduled/emergency surgery and whether the surgery involved cardiopulmonary bypass). TIA, transient ischaemic attack.

**Figure 4:**
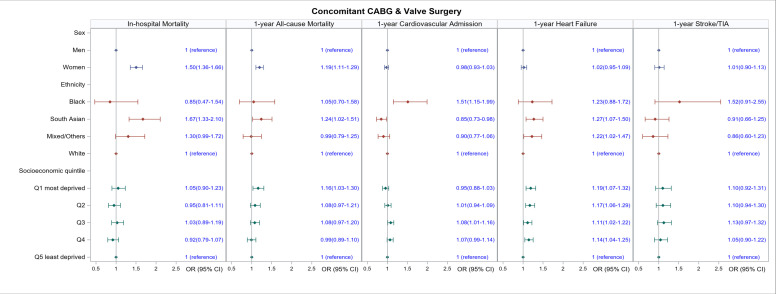
Association of sex, ethnicity and socioeconomic quintile with in-hospital mortality and 1-year outcomes for people undergoing concomitant coronary artery bypass grafting (CABG) and valve surgery. All models are adjusted for age, Charlson index, frailty status, year of surgery and operative characteristics (scheduled/emergency surgery and whether the surgery involved cardiopulmonary bypass). TIA, transient ischaemic attack.

#### Mid-term and long-term outcomes

At 1 year, mortality and hospital readmissions decreased slightly (by 8% and 9%, respectively) across the two time periods (2010–2014 and 2015–2019) (table 2). Mortality was higher in people undergoing valve surgery than CABG (7.6% valve surgery vs 3.8% CABG in 2010–2014, 7.5% vs 3.4% in 2015–2019). By 5 years, mortality had generally trebled across all types of surgery (to between 13% and 27%, 2010–2014).

In 1 year, readmission for any cardiovascular cause was higher in people undergoing valve surgery than CABG (25.4% vs 17.3% in 2010–2014 and 23.4% vs 15.5% in 2015–2019). By 5 years, readmission rates had more than doubled across all types of surgery, with between 35% and 44% of all people undergoing cardiac surgery having a readmission (2010–2014).

Crude mortality and hospital readmission rates at 1 year were generally higher in women compared with men across all types of surgery (online supplemental table S6). Mortality differences persisted for all outcomes after adjustment in women who had CABG (figure 2) and combined CABG and valve (figure 4) who had a 24% and 19% increased odds of death (OR 1.24, 95% CI 1.16 to 1.33 and OR 1.19, 95% CI 1.11 to 1.29), respectively, compared with men. Women who had CABG surgery had increased odds of hospital readmission (OR 1.12, 95% CI 1.08 to 1.16) compared with men, but this was not the case for women who had valve surgery (figures3  4).

Crude 1-year mortality and hospital readmission were generally higher in black people compared with white people across all types of cardiac surgery (online supplemental table S6). After adjustment, there were no significant differences in 1-year mortality between black and white people, although black people who had CABG and valve surgery had 31% and 33%, respectively, higher odds of being readmitted to hospital for heart failure (OR 1.31, 95% CI 1.08 to 1.58 and OR 1.33, 95% CI 1.16 to 1.53, respectively); and black people who had valve and combined CABG and valve surgery had a 14% and 51% higher odds, respectively, of being readmitted to the hospital for any cardiovascular cause (OR 1.14, 95% CI 1.02 to 1.28 and OR 1.51, 95% CI 1.15 to 1.99, respectively).

South Asians undergoing isolated CABG and valve surgery had similar 1-year mortality as white people(figures2  3), but those undergoing CABG and valve surgery had higher odds of dying at 1 year (OR 1.24, 95% CI 1.02 to 1.51). Compared with white people, South Asians had slightly lower odds of readmission for any cardiovascular cause (CABG: OR 0.90, 95% CI 0.85 to 0.96, valve surgery: OR 0.87, 95% CI 0.79 to 0.96; combined CABG and valve surgery: OR 0.85 95% CI 0.73 to 0.98) but higher odds of readmission for heart failure (OR 1.27, 95% CI 1.07 to 1.50).

The odds of dying increased with increasing levels of deprivation (figures2^4^). Across all types of surgery, people in the most deprived quintiles had an 18% (CABG), 10% (valve) and 16% (combined CABG and valve) increased odds of dying at 1 year compared with people in the least deprived quintile (OR 1.18, 95% CI 1.07 to 1.30; OR 1.10, 95% CI 1.01 to 1.18; OR 1.16, 95% CI 1.03 to 1.30, respectively). Similarly, the odds of heart failure readmission increased with increasing levels of deprivation: CABG by 22% (OR 1.22, 95% CI 1.14 to 1.31); valve surgery by 23% (OR.1.23, 95% CI 1.16 to 1.31); combined CABG and valve surgery by 19% (OR 1.19, 95% CI 1.07 to 1.32).

## Discussion

There has been a decline in the use of cardiac surgery as a treatment modality over time, a pattern observed in both Europe^1^ and the USA.^11^ The increased risk profile of people undergoing cardiac surgery with the concurrent decrease in in-hospital mortality reflects the improved safety and quality of cardiac surgery.^1^ However, mid-term (1 year) outcomes (mortality and readmission) have not seen the same proportional improvement. Across all types of cardiac surgery, one in five people are readmitted to hospital within 1 year of surgery, rising to almost one in four for valve surgery.

This suggests that the biggest improvements to care have been in the in-hospital surgical pathways rather than ‘joined-up’ care. Cardiac surgery is often used as an example of an efficient patient-centred care pathway that achieves better outcomes than many other types of major surgery,^12^ yet, it is clear from these data that much remains to be done in implementing a complete model of care that optimises both the pre-operative selection and optimisation of patients and the postoperative period after hospital discharge.

We observed disparities in access to surgery and outcomes of surgery by demographic and socioeconomic characteristics, with women, people from deprived backgrounds and people of black ethnicity being less likely to be offered surgery and experiencing poorer outcomes. For deprivation, there was an almost linear pattern of decreasing odds of getting surgery with increasing levels of deprivation, even after adjustment for multiple covariates, including frailty and comorbidities. From an intersectional perspective, the disparities widened when more than one inequality factor was considered; for example, women from deprived areas were less likely to get surgery than men from deprived areas, while black women were less likely to get surgery than black men.

These patterns of inequality in the NHS have been consistently observed across many types of treatment, including aortic valve replacement,^13^ orthopaedic surgery^14^ and the elective backlog in England.^15^ NHS England’s National Healthcare Inequalities Improvement Programme highlighted multiple reasons for these findings, including availability of services in local areas, access to transport, language and literacy barriers, negative past experiences, misinformation and fear.^16^

The reasons for the differences in outcomes between women and men after cardiac surgery, in particular CABG, have been extensively discussed.^12^ Compared with men, women have delayed diagnosis, more comorbidities, a broader array of symptoms,^17^ more unstable or acute presentation, a higher rate of small vessel disease and reduced patient and clinician perception of actual risk.^12 18 19^ Some of these factors also likely apply to black people and people from deprived backgrounds.^20 21^

Ethnic inequalities in the NHS have been attributed to poor education, social status and poverty outside the health system. Inside the health system, there may be poor quality or discriminatory treatment from healthcare staff, a lack of high-quality recorded ethnic monitoring data; a lack of appropriate interpreting services^22^ and avoidance of seeking help for health problems due to fear of racist treatment from healthcare professionals. While in the USA, one of the key mechanisms of ethnic inequalities in cardiac surgery has been purported to be lack of access to high-quality hospitals for black ethnic groups,^23^ this is not true in the UK. Cardiac surgery is a specially commissioned, nationally funded service and the vast majority of cardiac surgery takes place in these regional centres. While clustering of ethnic groups within particular centres may occur, these are often high-volume, high-quality centres—the causes are clearly wider than poor-quality care alone.

There is an urgent need to address inequalities through enhanced data linkage and improved transparency and publication of data from benchmarking exercises on inequality characteristics and ensuring equity of the workforce and pathways people use to access care. The NHS Long Term Plan has set out priority areas, urgent actions and several initiatives (eg, Core20PLUS5) for tackling health inequalities in the NHS in the next decade.^15^

## Limitations

A key limitation of using HES is that it relies on diagnostic and procedure codes for identifying individuals and their outcomes. Coding practices vary between hospitals, particularly for diagnostic coding and although the accuracy of coding has improved markedly over the past 20 years^24^, and caution is needed when interpreting time series of HES data. However, our findings, in terms of trends in cardiac surgery, increased complexity of people presenting for surgery and short-term (in hospital) outcomes mirror findings from the National Adult Cardiac Surgery Audit,^1^ which uses data input directly by clinicians, therefore our data are likely to be broadly accurate.

Another limitation is that over 10% of people within the HES database have an unknown ethnicity,^25^ therefore, our findings with regard to ethnicity need to be interpreted with caution. We did not attempt to impute missing ethnicity data, largely because the value of this analysis is uncertain given that ethnicity data are not likely to be missing at random and there are likely to be fundamental differences between the population with and without ethnicity status. Also, the ‘mixed/other’ ethnic group is heterogeneous and cannot be meaningfully interpreted because it includes people of mixed race and specific ethnic groups (eg, Chinese) for which numbers were too small to analyse separately. Furthermore, ethnicity data quality is further affected by inconsistent use of ethnicity codes and systematic biases that may affect records for minority ethnic people disproportionately. As such, there is the potential for selection and information bias.

We used people admitted to hospitals with IHD and HVD as our denominators for CABG and valve surgery rates. These populations do not necessarily represent the population ‘at-risk’ of having cardiac surgery—there are likely to be underlying differences in eligibility for surgery based not only on demographics and comorbidities (for which we adjusted) but also on pathology (eg, greater proportion of degenerative valve disease in the admitted population vs the valve surgery population) and other elements of operative risk scores (eg, EuroSCORE II^26^/STS risk score^27^) which are not collected in HES. Furthermore, the valve surgery population is highly heterogeneous, encompassing different types of valve surgeries and pathologies which may influence outcomes differently.

## Conclusions

The improvements in in-hospital outcomes after cardiac surgery that have been so well documented over the past decade do not translate into a similar improvement in mid-term and long-term outcomes. While for some groups of people undergoing cardiac surgery, both absolute and relative inequalities have decreased, this pattern is not consistent. These are stark in the case of black people, South Asians and women who are consistently more likely to die after heart surgery than their white and male peers. Targeted interventions are required across the cardiovascular medicine pathway to ensure that interventions are applicable to and implemented in underserved populations. Healthcare providers must also undertake surveillance using routinely collected data to ensure that their interventions are reaching underserved groups.

## supplementary material

10.1136/heartjnl-2024-324292 online supplemental file 1

## Data Availability

No data are available.
